# Effects of nitrogen and tiller type on grain yield and physiological responses in rice

**DOI:** 10.1093/aobpla/plx012

**Published:** 2017-03-30

**Authors:** Yang Wang, Jianwei Lu, Tao Ren, Saddam Hussain, Chen Guo, Sen Wang, Rihuan Cong, Xiaokun Li

**Affiliations:** 1 Key Laboratory of Arable Land Conservation (Middle and Lower Reaches of Yangtze River), Ministry of Agriculture, Microelement Research Centre, College of Resources and Environment, Huazhong Agricultural University, No.1 Shizishan Street, Hongshan District, Wuhan 430070, China; 2 Department of Agronomy, University of Agriculture, Jail Road, Faisalabad, Punjab 38040, Pakistan

**Keywords:** Chlorophyll fluorescence, nitrogen, nitrogen harvest index, rice, senescence, tiller, vascular bundle

## Abstract

The increasing food demands from an expanding population necessitate global efforts to increase crop production and ensure food security. The rate of nitrogen (N) fertilizer application is strongly related to crop yield. However, although the application of N fertilizer significantly increases the number of tillers in rice, late emerging tillers usually produce lower yields compared with early emerging tillers. Understanding the physiological constraints of late emerging rice tillers is critical for further increasing rice grain yields. Two-year field experiments, consisting of four nitrogen fertilizer levels, were conducted in order to study variations in the physiological characteristics of different types of tillers. The results revealed that the contributions of late emerging tillers to population rice grain yields improved with increased N levels. However, spikelets per panicle and the grain filling of late emerging tillers were significantly lower than that of the main stems or early emerging tillers under all N levels. The nitrogen harvest index of late emerging tillers was lower than that of main stems and early emerging tillers, and differences gradually increased under higher N rates. Nutrient source deficiency was a primary factor for the low productivity of late emerging tillers. Additionally, rapid malondialdehyde accumulation and delayed emergence determined the short growth duration of late emerging tillers. Further, low actual photochemistry efficacy (Φ_PSII_) resulted in insufficient photosynthetic assimilate supply in late emerging tillers, whereas highly constitutive non-photochemical energy dissipation (Φ_NO_) might damage the photosynthetic system. Moreover, the low activity of SuSase and spikelets per panicle revealed both inadequate sink activities and storage sites. The identification of these limiting factors in late emerging rice tillers will assist in closing the ‘yield gap’ between late emerging tillers and early emerging tillers, and contribute to further increasing rice grain yields.

## Teaser

Tillering is an important agronomic trait for rice population quality and grain production. Nitrogen fertilizer application increased the number of rice tillers, but not every tiller contributes equally to the overall yield. Heterogeneity in tiller yield of rice increased with increasing nitrogen levels. Typically, late emerging tillers contributed less to the grain yield. Under high nitrogen conditions, most of the nutrients were retained in the straw of late emerging tillers; these tillers have high transport efficiency in vascular bundles, but there is less time for nutrients to transport into the grain. Despite the high light intensity, less than half of the light is used for photosynthesis in late emerging tillers. The low activity of enzymes related to grain filling in late emerging tillers is another reason for low yield.

## Introduction

Branching comprises a protective mechanism for higher plants, by assisting with reducing the impacts of injury, and facilitating adaptation to the environment (Horvath *et al.* 2003). Gramineous plants have a special type of side shoot referred to as a tiller. which is distinct from the lateral branching of dicotyledonous plants. The continual emergence of new tillers takes place from the main stem and these generate their own roots to grow independently, hence ensuring the survival of the gramineous plants under diverse conditions (Cybopoba 1959). Moreover, the number of tillers has been reported to have a positive association with plant biomass and economic yields in rice (Deng *et al.* 2015), oat (Deiss *et al.* 2014), wheat and barley (Alzueta *et al.* 2012). Rice is one of the most important agricultural commodities in the world. From 1980 to 2016, dedicated rice planting areas were increased by only 12 % worldwide, while total rice production increased by 78 % (USDA 2016). This indicates that strategies for increasing rice yields per unit area were the key driving force behind increased rice production. Nevertheless, the amount of arable land that is available for rice production faces a number of serious challenges, including rapid urbanization, land disruption and environmental degradation (Deng *et al.* 2015). Under the scenario of limited cultivated land resources, it is critical to ensure future food security through continuous improvements in grain yields per unit area (Peng *et al.* 2008).

Tillering is an important agronomic trait for rice population quality and grain production (Counce and Wells 1990; Ling, 2000). This is because excess tiller production results in a dense canopy, which provides a moist micro-environment favourable for diseases and pests, whereas too few tillers results in insufficient numbers of panicles (Cu *et al.* 1996). Under field conditions, the application of nitrogen (N) fertilizer is the most common and effective way to enhance the tiller population, as it increases the cytokinin content within tiller nodes and further enhances the germination of the tiller primordium (Liu *et al.* 2011). Additionally, N evokes a significant effect on the promotion of tiller development (Sakakibara *et al.* 2006). Although many tillers may be generated subsequent to a sufficient supply of N, not every tiller contributes equally to the overall yield. Typically, late emerging tillers do not contribute significantly to the grain yield of rice (Wang *et al.* 2007); however, theoretically, they possess the potential for high productivity, due to the totipotency of rice coleoptile tissue (Oinam and Kothari 1995). Rice requires N in larger quantities than any other nutrient, and it is the most critical limiting factor that influences grain yields (Siddiqui *et al.* 2008), but no obvious decline in straw N concentration was discovered in late emerging tillers (Sparkes *et al.* 2006; Wang *et al.* 2016). Hence, elucidating the adverse effects on N accumulation and transport may assist in identifying the mechanisms underlying the low production of late emerging rice tillers.

Mohapatra and Kariali (2008) indicated that short growth duration was the principal reason for poor grain production by late emerging rice tillers. However, limited information is available in regard to whether the negative effects of an insufficient growth stage on grain filling, and consequently on grain yields, may be offset by delaying harvesting. A reduced tolerance to photo-oxidative stress has been put forward as an additional factor by Kariali *et al.* (2012). Recently, Wang *et al.* (2016) reported that the unequal distribution of photosynthetically active radiation (PAR) was the source of heterogeneity in individual tiller’ yields, in that early emerging superior tillers pre-empted the uppermost light source, and shaded the late emerging tillers under limited light conditions.

For this study, the tillers were divided into different types (main stem, early emerging tillers and late emerging tillers) according to the time of emergence of individual tillers under field conditions. The specific objectives were (i) to compare the yield performance of different types of tillers under contrasting N availability levels; and (ii) to determine the responses of physiological traits in different types of tillers to variable N applications.

## Methods

### Site description

Field experiments were conducted at two adjacent fields located in Wuxue County (30°11′ N 115°59′ E), Hubei Province, in Central China, during the 2014 and 2015 rice growing seasons. The soil type was hydromorphic paddy soil, which is a silty clay loam derived from quaternary yellow sediments. Prior to experimentation, soil samples extracted from the upper 20 cm layer were collected for chemical analyses. In 2014/2015, the soil pH, organic matter, total N content, available P and available K were 6.06/5.62, 23.4/29.4 g kg^−1^, 1.43/1.67 g kg^−1^, 14.2/13.3 mg kg^−1^ and 101.9/109.8 mg kg^−1^, respectively.

### Plant materials

Two *indica* hybrid varieties ‘Fengyuanyou-299’ and ‘C-Liangyouhuazhan’ were grown in 2014 and 2015, respectively. Both of these rice varieties are widely grown in the Yangtze River area, China. Prior to formal study, a preliminary field experiment was conducted in 2013, where two varieties ‘Liangyou-287’ and ‘Fengyuanyou-299’ were planted to observe the phenomenon of heterogeneity in rice tillers. In the preliminary experiment, the number of tillers and grain yields of different tillers were quantified (Table 1). Two-way ANOVAs showed that the yields of different types of tillers were significantly affected by genotype rather than the environment (Table 2). Repeatability statistics of Fengyuanyou-299 were included to verify that the phenotype data was reliable (Table 3).

**Table 1.** Number of tillers and grain yield of different types of tillers grown under various N application rates in 2013 and 2014. M, grain yield of main stem; E, grain yield of early emerging tiller; L, grain yield of late emerging tiller; M-E, grain yield of main stem minus grain yield of early emerging tiller; M-L grain yield of main stem minus grain yield of late emerging tiller; E-L grain yield of early emerging tiller minus grain yield of late emerging tiller. LY287, Liangyou 287; FYY299, Fengyuanyou 299. Different letters in the same column represent significant differences (*P* < 0.05) among different tiller types at the same N rate.

Year	Variety	N rate (kg ha^−1^)	No. of tiller (plant^−1^)	M	E	L	M-E	M-L	E-L
				(g tiller^−1^)					
2013	LY287	0	5.0 b	3.70 a	3.31 a	1.82 ab	0.39 a	1.88 b	1.48 b
		82.5	6.3 b	4.32 a	3.87 a	2.19 a	0.45 a	2.13 ab	1.68 ab
		165	8.5 a	4.25 a	3.94 a	1.42 b	0.30 a	2.83 a	2.52 a
		247.5	8.7 a	4.14 a	3.83 a	1.51 b	0.31 a	2.64 a	2.33 ab
	FYY299	0	4.5 c	5.54 a	5.24 a	3.05 a	0.30 a	2.50 b	2.19 b
		82.5	5.8 c	6.03 a	5.59 a	2.42 ab	0.44 a	3.62 ab	3.18 ab
		165	7.3 b	6.04 a	5.30 a	1.74 b	0.74 a	4.30 a	3.56 a
		247.5	9.3 a	5.67 a	5.13 a	1.70 b	0.54 a	3.97 ab	3.43 a
2014	LY287	0	3.8 c	3.21 c	2.70 c	1.62 a	0.51 a	1.59 c	1.08 b
		82.5	5.2 b	4.08 b	3.47 b	1.55 a	0.60 a	2.53 bc	1.92 ab
		165	6.3 a	4.17 b	3.95 ab	1.32 a	0.22 a	2.84 ab	2.63 a
		247.5	7.2 a	5.09 a	4.31 a	1.25 a	0.79 a	3.84 a	3.05 a
	FYY299	0	4.3 c	5.64 a	5.15 a	3.42 a	0.49 a	2.22 b	1.73 b
		82.5	6.2 bc	6.44 a	5.78 a	3.08 ab	0.66 a	3.36 ab	2.70 ab
		165	6.8 b	6.48 a	5.87 a	1.90 ab	0.62 a	4.59 a	3.97 ab
		247.5	9.0 a	6.44 a	5.87 a	1.34 b	0.57 a	5.10 a	4.53 a

**Table 2.** Results of two-way analyses of variance (ANOVA) for analyses of differences in number of tillers, grain yield of different types of tillers and their yield gap to examine the effects of ‘genotype’ and ‘environment’ for rice. M, grain yield of main stem; E, grain yield of early emerging tiller; L, grain yield of late emerging tiller; M-E, grain yield of main stem minus grain yield of early emerging tiller; M-L grain yield of main stem minus grain yield of late emerging tiller; E-L grain yield of early emerging tiller minus grain yield of late emerging tiller. G, genotype; E, environment, indicate different years. Values with P < 0.05 are in boldface.

Source	df	No. of tiller		M		E		L		M-E		M-L		E-L	
		*F*	*P*	*F*	*P*	*F*	*P*	*F*	*P*	*F*	*P*	*F*	*P*	*F*	*P*
G	1	1.4	0.239	131.7	**<0.001**	133.6	**<0.001**	15.2	**<0.001**	1.3	0.263	23.3	**<0.001**	18.1	**<0.001**
E	1	11.7	**0.001**	1.9	0.172	0.5	0.488	0.1	0.816	1.9	0.174	1.3	0.262	0.4	0.548
G×E	1	7.5	**0.009**	1.4	0.243	2.3	0.134	1.7	0.193	0.3	0.621	0.1	0.82	0.0	0.962
Error	8	–	–	–	–	–	–	–	–	–	–	–	–	–	–

**Table 3.** Repeatability (r_e_) of number of tillers and grain yield of different types of tillers in variety FYY299 during 2013–2014. M, grain yield of main stem; E, grain yield of early emerging tiller; L, grain yield of late emerging tiller; M-E, grain yield of main stem minus grain yield of early emerging tiller; M-L, grain yield of main stem minus grain yield of late emerging tiller; E-L, grain yield of early emerging tiller minus grain yield of late emerging tiller. *r_e_* ≥ 0.6 indicate high repeatability; 0.3 < *r_e_* < 0.6 indicate medium repeatability; *r_e_* ≤ 0.3 indicate low repeatability.

N rate (kg ha^−1^)	No. of tiller	M	E	L	M-E	M-L	E-L
0	0.82	0.53	0.33	0.02	0.13	0.36	0.33
82.5	0.45	0.06	0.71	0.74	0.85	0.74	0.87
165	0.34	0.27	0.11	0.96	0.47	0.85	0.74
247.5	0.38	0.62	0.47	0.36	0.88	0.45	0.34
Mean	0.50	0.37	0.41	0.52	0.58	0.60	0.57

In the 2014 experiment, pre-germinated seeds were sown in a seedbed on 25 June, and the resulting seedlings were transplanted on 20 July at a hill spacing of 0.167 m × 0.200 m, with a single seedling per hill. In the 2015 experiment, pre-germinated seeds were sown in a seedbed on 25 May, and the seedlings were transplanted on 23 June at a hill spacing of 0.175 m × 0.214 m, with a single seedling per hill. In order to prevent seepage and nutrient flow, each plot was separated with 0.2-m-wide bunds, which were covered with a double layered plastic film (at a 0.3 m soil depth). Flooding was maintained in the field during transplantation until 10 days prior to crop maturity. Weeds, diseases, birds and insects were intensively controlled during the entire growing season to avoid yield losses in both years.

### Experimental design

The study was laid out in a randomized complete block design with three replications. Treatments comprised of four N application rates: (i) N_0_ (no N fertilizer application); (ii) N_82.5_ (82.5 kg N ha^−1^); (iii) N_165_ (165 kg N ha^−1^, which is the recommended rate for rice in Hubei province, Wang *et al.* 2012) and (iv) N_247.5_ (247.5 kg N ha^−1^). Nitrogen (urea) was applied as 50 % at the basal stage, 25 % at the tillering stage (15 days after transplanting; DAT) and 25 % at the panicle initiation stage (40 DAT). Phosphorus, in the form of calcium superphosphate (75 kg P_2_O_5_ ha^−1^), and zinc, in the form of zinc sulphate heptahydrate (5 kg Zn ha^−1^), were applied as basal dose. Potassium (potassium chloride) was applied at 75 kg K_2_O ha^−1^, 70 % at the basal stage and 30 % at the panicle initiation stage.

### Tiller type and yield components

For the determination of tiller order, the main stems (M) of 15 randomly selected plants from each plot were labelled with small plastic tags, while other tillers were tagged according to the emerging tiller order. At the panicle mature stage, six hills were sampled, and the grain yield and yield components of individual stem/tillers were measured. The panicles were subsequently hand-threshed, and the filled spikelets, unfilled spikelets, and grain weight were quantified using a seed analysis instrument (SC-G, Wanshen Detection Technology Co., Ltd., Hangzhou, China). Except for the main stems, tillers under the same treatments were divided into early emerging tillers (the first half) and late emerging tillers (the second half) based on their emergence time. In order to study, the physiological differences among different types of tillers during their growth duration, the first and second emerging productive tillers were regarded as early emerging tillers (E), while the last two emerging productive tillers were denoted as late emerging tillers (L).

### Transport load of grain filling

At the heading stage, four tagged tillers of different types in each treatment were selected and the culm was cut at 5 cm below the panicle neck. The first internode at the top of the stem was fixed with FAA liquid (70 % ethyl alcohol 90 mL, glacial acetic acid 5 mL, formalin 5 mL and glycerinum 5 mL) for 24 h at 4 °C, and then stored in 15 % (v/v) hydrofluoric acid for 25 days (desiliconization). The paraffin section process was based on a method described in a previous study (Li 2009). The anatomical structures of the first internode were observed and photographed via a microscope (Nikon eclipse 80i, Japan). The area of the vascular bundle (VB) was quantified using Image-pro plus 6.0 software (Media Cybernetics, Bethesda, MD, USA).

To determine the N accumulation rate of the grain, eight tagged panicles of different tiller types from each plot were sampled every 10 days. The grains of the sampled panicles were oven dried at 105 °C for 30 min and then at 75 °C until they were at a constant weight. The grains were then combined, ground to pass through a 1-mm mesh screen, and then digested by H_2_SO_4_ and H_2_O_2_ (Bao 2000). The N content of the digested samples was determined using an automated continuous flow analyser (Seal, Norderstedt, Germany). The grain N accumulating rate (*G*) was calculated using the equation:

$$G\;\text{=}\;{(N}_{t}-N_{i})/T$$

where *G* is the grain N accumulating rate (mg d^−1^); N_i_ and N_t_ are the initial and terminal N accumulations during grain filling, respectively (mg), and *T* is the grain filling time (d).

For a further comparison of N flow efficiency among different tillers, a new concept referred to as ‘load’ was utilized to represent the level of transport efficiency. We then normalized the value of load by dividing it by maximum load for all treatments. The normalized load was given as:

Load = G/VB area

$$\text{Normalized}\;{\text{Load=Load}}_{i}{/\text{Load}}_{\text{max}}$$

where Load is the transport efficiency of the first internode of the stem (mg d^−1^ μm^−2^); *G* is the grain N accumulating rate (mg d^−1^); VB area is the summed area of large and small vascular bundles (μm^2^); Load_i_ is the load of any treatment; Load_max_ is the maximum of load for all treatments.

The N transport efficiency, from source organ to sink organ, was expressed as the nitrogen harvest index (NHI, Grain N accumulation/Plant N accumulation).

### Chlorophyll fluorescence measurements

Four tagged tillers of different types in each plot were selected, and the flag leaves were used to measure the fluorescence parameters with a portable photosynthesis apparatus that was coupled with a 6400–40 leaf chamber at the grain filling stage. In the morning (2:00–3:00 am), the initial fluorescence yield (*F_o_*) was measured in a dark-acclimated state, followed by a saturating pulse in order to measure the maximum fluorescence yield (*F_m_*).

In the late morning (9:00–11:00 am), the minimum chlorophyll fluorescence yield (*F_o_*), steady-state chlorophyll fluorescence (*F_s_*) and maximum fluorescence ($F_{m}^{\prime }$) were recorded in a light-acclimated state. Photo chemical quenching (qP) and non-photochemical quenching (NPQ) were estimated according to Bilger and Björkman (1990).

$$\text{qP=}(F_{m}^{\prime }-F_{s})/(F_{m}^{\prime }-F_{o}^{\prime }),\text{ NPQ=}(F_{m}-F_{m}^{\prime })/F_{m}^{\prime }$$

Quantum efficiency of PSII photochemistry was estimated (Genty *et al.* 1989) as;

$$\Phi_{\text{PSII}}\text{=}(F_{m}^{\prime }-F_{s})/F_{m}^{\prime }$$

The fraction of still open PSII reaction centres (qL) was measured as;

$$\text{qL=qP×}(F_{o}^{\prime }/F_{s})\;$$

Within the leaf chamber, the leaf temperature was maintained at 30 °C, the photosynthetic photon flux density was maintained at 1200 μmol m^−2^ s^−1^, and the CO_2_ concentration was set at 400 µmol mol^−1^ during measurements.

The allocation of photons that were absorbed by the PSII antennae for photosynthetic electron transport and thermal dissipation was assessed by defining the actual photochemical efficiency (Φ_PSII_), regulated thermal dissipation (Φ_NPQ_; Genty *et al.* 1996) and constitutive non-photochemical energy dissipation (Φ_NO_; Genty *et al.* 1996). The Φ_NPQ_ and Φ_NO_ were calculated according to Kramer *et al.* (2004).

$$\Phi_{\text{NO}}\text{=}1/(\text{NPQ+}1\text{+qL×}(F_{m}/F_{o}-1));$$

$$\Phi_{\text{NPQ}}\text{=}1-\Phi_{\text{PSII}}-\Phi_{\text{NO}}.$$

### Plant biochemical analyses

The leaves and grains of samples were immediately frozen in liquid nitrogen and then stored at –80 °C to determine the physiological indices. The level of leaf senescence was determined by measuring the amount of malondialdehyde (MDA), a product of lipid peroxidation, following the method of Wang and Huang (2015). Fresh material (about 0.5 g) was homogenized in 5 mL of 10 % (w/v) cold trichloroacetic acid (TCA). The homogenized mixture was centrifuged at 8000 rpm for 10 min at 4 °C and 2 mL of thiobarbituric acid (TBA) reagent (0.5 % TBA in 10 % TCA) was added to a 2 mL aliquot of the supernatant. The mixture was heated in boiling water for 20 min and cooled rapidly in an ice bath. After centrifugation at 3000 rpm for 10 min, the absorbance was recorded at 532 nm, 600 nm and 450 nm. The concentration of MDA was calculated as:

$$\text{MDA}(\text{μmol}/L)\text{=}6{.45\text{×}(\text{OD}}_{532}-{\text{OD}}_{600})-0{.56\text{×OD}}_{450}$$

The method for preparation of enzyme extracts was modified from Yang *et al.* (2003). Activity of sucrose synthetase (SuSase) was assayed in the cleavage direction and analysed using the modified method of Ranwala and Miller (1998). The reaction mixture contained 100 mM HEPES-NaOH (pH 7.5), 50 mM sucrose, 5 mM UDP, 5 mM magnesium acetate and 5 mM DTT. The buffer (0.8 mL) was pre-incubated in a water bath at 30 °C for 5 min prior to the addition of 0.2 mL of enzyme extract to initiate the reaction. The standard reaction time was 30 min, and the assay was linear within the standard time and enzyme volume used. Following incubation, the reaction was terminated by placing the reaction mixture in a boiling water bath for 5 min. Controls for the assay consisted of reactions that were carried out with inactivated (boiled for 5 min) enzyme. After natural cooling, 0.5 mL 3,5-dinitrosalicylic acid solution (DNS) was added to reaction mixture to determine the fructose produced. The mixture was introduced into a boiling water bath for exactly 5 min, and then immediately cooled in ice, followed by the addition of 3.5 mL deionized water. After fully mixing, the absorbance was recorded at 540 nm.

Protein concentrations were determined via the method described by Bradford (1976), using bovine γ-globulin as a standard.

### Statistical analyses

Data were statistically analysed using SPSS for Windows (Version 19.0, Chicago, IL, USA). All data were previously tested for normality using the Shapiro-Wilk method and homoscedasticity using a Levene test. Where appropriate, the data were log_10_ transformed to meet the assumption of homogeneity of variance and normality. In cases where the ANOVA assumptions continued to be violated following data transformation, treatment differences were assessed using the more conservative Kruskal–Wallis nonparametric test. When variances of data were homogeneous, one-way ANOVA was used to determine differences among yield, yield components, number of VB, area of VB, photosynthetic parameters and activity of SuSase per tiller type only. For all analyses, the significance level was set at *P *< 0.05. Two-way ANOVA was used to test the significance of main effects (N level and tiller type) and their interaction with the above-mentioned parameters. The figures were plotted using the Origin 8.0 software program (Microcal Software, Northampton, MA).

## Results

### Yield and yield components of different types of tillers

Two-way ANOVAs showed that the number of panicles per square meter, number of spikelets per panicle and yields per hectare were significantly affected by nitrogen level (N) and tiller type (T) in 2014 and 2015 (Table 4). Also, the effects of N and T interactions (N × T) on panicles and yields were highly significant. The grain filling percentage was only significantly affected by T. For the grain weight, the N × T effect was not significant, while N and T effects were significant (with the exception of T in 2015).

**Table 4.** Results of two-way analyses of variance (ANOVA) for analyses of differences in panicles, spikelets, grain filling percentage, grain weight and yield per hectare to examine the effects of ‘nitrogen’ and ‘type’ for rice. Values with P < 0.05 are in boldface.

Source	df	Panicles		Spikelets		Grain filling		Grain weight		Yield	
		*F*	*P*	*F*	*P*	*F*	*P*	*F*	*P*	*F*	*P*
2014											
Nitrogen (N)	3	41.9	**<0.001**	4.3	**0.015**	4.1	0.018	3.7	**0.026**	32.9	**<0.001**
Type (T)	2	164.7	**<0.001**	282.4	**<0.001**	13.0	**<0.001**	6.2	**0.007**	128.3	**<0.001**
N×T	6	10.7	**<0.001**	2.0	0.099	1.6	0.197	2.0	0.102	8.6	**<0.001**
Error	24	–	–	–	–	–	–	–	–	–	–
2015											
N	3	76.4	**<0.001**	7.2	**0.001**	2.6	0.073	7.3	**0.001**	106.3	**<0.001**
T	2	513.9	**<0.001**	145.6	**<0.001**	5.9	**0.008**	0.5	0.624	549.4	**<0.001**
N×T	6	19.8	**<0.001**	0.3	0.920	0.5	0.813	0.2	0.957	23.4	**<0.001**
Error	24	–	–	–	–	–	–	–	–	–	**–**

Rice tillers were divided into early emerging tillers and late emerging tillers in accordance with their emergence times. Typically, the main stems possessed the highest number of spikelets per panicle, grain filling percentage and grain weight, followed by the early emerging tillers; the poorest yields were produced by the late emerging tillers under all N treatments (Table 5). In comparison with no-N treatment, the number of spikelets per panicle was increased by 7.2 % in the main stems, 4.9 % in early emerging tillers, and 4.6 % in late emerging tillers, following the application of N in 2014. Likewise in 2015, the application of N increased the number of spikelets per panicle in main stems (10.5 %), early emerging tillers (8.5 %) and late emerging tillers (11.0 %). The grain filling percentage and grain weight of the same types of tillers were less affected by N levels.

**Table 5.** Yield and yield components on different types of rice tiller populations grown under various N application rates in 2014 and 2015. M, main stem; E, early emerging tiller; L, late emerging tiller. Different letters in the same column represent significant differences (*P* < 0.05) among different tiller types under the same N rate.

N rate (kg N ha^−1^)	Tiller type	Panicles (m^−2^)	Spikelets (panicle^−1^)	Grain filling (%)	Grain weight (mg)	Yield (t ha^−1^)	Yield contribution (%)
2014							
0	M	30 b	236 a	85.0 a	27.2 a	1.67 b	27.6
	E	55 a	209 a	87.4 a	27.5 a	2.83 a	46.8
	L	45 ab	146 b	84.2 a	27.2 a	1.55 b	25.6
	Mean	43	197	85.5	27.3	2.02	–
82.5	M	30 c	247 a	91.5 a	27.7 a	1.90 b	21.6
	E	85 a	204 b	87.2 ab	28.1 a	4.36 a	49.7
	L	70 b	158 c	81.6 b	27.6 a	2.52 b	28.7
	Mean	62	203	86.8	27.8	2.93	–
165	M	30 b	248 a	90.3 a	28.7 a	1.96 c	20.0
	E	90 a	225 a	85.6 b	28.1 a	4.90 a	49.9
	L	85 a	151 b	81.9 c	27.6 a	2.96 b	30.1
	Mean	68	208	85.9	28.1	3.27	–
247.5	M	30 c	264 a	84.6 a	28.7 a	1.94 c	15.8
	E	120 a	229 b	82.8 ab	28.2 a	6.75 a	55.0
	L	110 b	149 c	79.2 b	26.8 b	3.58 b	29.2
	Mean	87	214	82.2	27.9	4.09	–
2015							
0	M	27 c	235 a	93.7 a	23.7 a	1.39 b	22.0
	E	76 a	197 b	92.0 a	23.9 a	3.28 a	51.7
	L	54 b	145 c	90.3 a	23.9 a	1.67 b	26.3
	Mean	52	192	92.0	23.8	2.11	–
82.5	M	27 c	255 a	92.9 a	24.5 a	1.54 c	15.0
	E	112 a	210 b	91.0 a	24.4 a	5.14 a	53.2
	L	90 b	151 c	90.3 a	23.5 a	2.98 b	31.8
	Mean	76	205	91.4	24.1	3.22	–
165	M	27 b	275 a	94.5 a	23.8 a	1.65 c	13.4
	E	126 a	222 b	93.2 a	24.1 a	6.19 a	50.3
	L	117 a	172 c	92.4 a	24.2 a	4.47 b	36.3
	Mean	90	223	93.4	24.0	4.10	–
247.5	M	27 c	249 a	93.9 a	23.7 a	1.48 c	11.9
	E	144 a	209 b	92.9 ab	23.7 a	6.54 a	52.5
	L	130 b	160 c	91.2 b	23.6 a	4.44 b	35.6
	Mean	100	206	92.7	23.7	4.15	–

It is recognized that fertilization with N significantly increases rice population yields. For the same types of tillers, grain yields were increased under higher N application in both years (Table 5). Under N_0_ treatment, no obvious differences between the yields of main stems and late emerging tillers were observed. However, the application of N significantly increased the number of late emerging tillers, which resulted in higher yields from the late emerging tillers than those from the main stems. The application of N decreased the yield contribution of the main stems, while increasing the yield contributions of the late emerging tillers. The highest yield contribution came from early emerging tillers (46.8 %–55.0 % in 2014; 50.3 %–53.2 % in 2015) under all N fertilizer levels.

### Grain N accumulation and N harvest index

**Figure 1:**
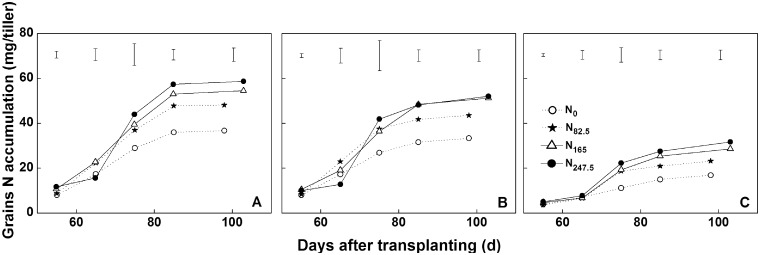
Dynamics of grain N accumulation in main stems (A), early emerging tillers (B) and late emerging tillers (C) grown under various N application rates in 2015. The vertical bars represent LSD at *P*** **=** **0.05.

N accumulation in grains increased during the filling stage, and the curve shape for different types of tillers at the same N levels was similar (Fig. 1). The application of N fertilizer increased the N accumulation in the grains, where the grain N uptake reached its peak at ∼15 days prior to harvest. Hence, the grain N accumulation rate (G) under high N treatments (N_165_ and N_247.5_) was higher than that under low N treatments (N_0_ and N_82.5_). Under identical N treatments, *G* was the highest in the main stem, followed by the early and late emerging tillers. Furthermore, a slow *G* was observed in the main stems and early emerging tillers under N_247.5_ treatment, which may have been due to excess N fertilizer application that acted to delay the vegetative growth stage.

**Figure 2:**
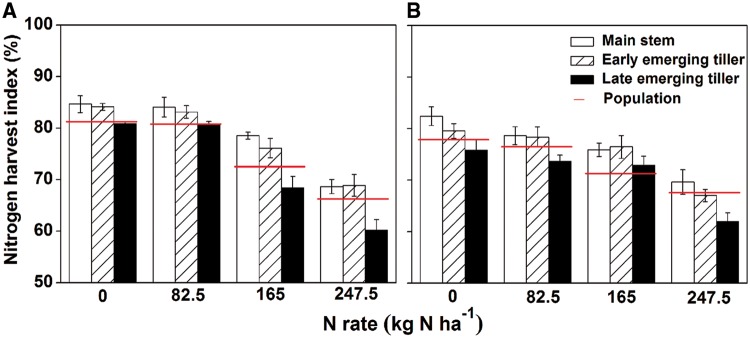
Changes in nitrogen harvest index (NHI) of different types of rice tillers under various N application rates in 2014 (A) and 2015 (B). The error bars indicate the SD.

Figure 2 indicates that the nitrogen harvest index (NHI) in the rice population declined with increasing N rates in 2014 and 2015. Considering the individual tiller, NHI in the same type of tiller decreased with an increase in N rates. At the same N levels, NHI in the late emerging tillers was lower than in the main stem and early emerging tillers, but no significant difference was observed between the main stem and early emerging tillers. Furthermore, the gap between early emerging tillers and late emerging tillers gradually grew with increasing N levels.

### Transport load of the first internode

**Figure 3:**
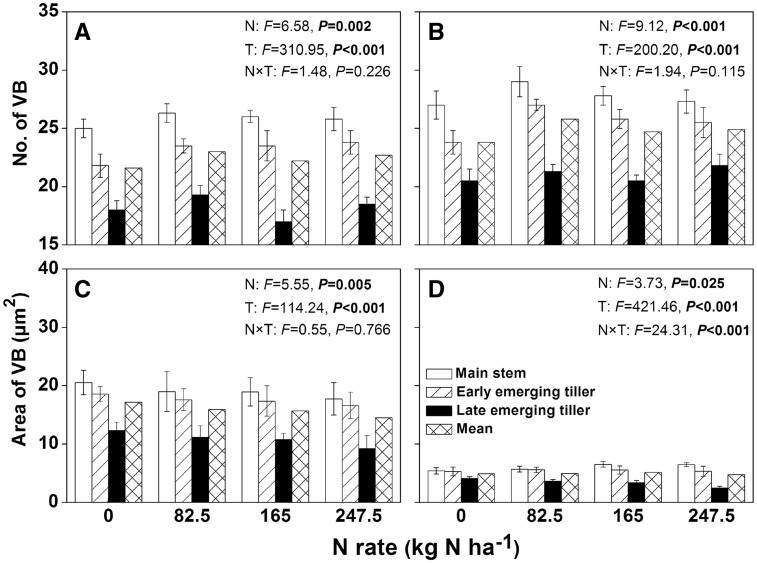
Number and area of large (A, C) and small (B, D) vascular bundles in the first internode of stem for different types of rice tillers grown under various N application rates in 2015. The df in Nitrogen (N), tiller type (T), N** **×** **T and error is 3, 2, 6 and 24, respectively. The error bars indicate the SD.

The first internode at the top of the rice stem provides the only channel for the transport of photosynthetic products of leaves to grains, and its development was significantly affected by the type of tiller. Two-way ANOVA indicated that the number of LVB (large vascular bundle) or SVB (small vascular bundle) and area of LVB or SVB were affected by N levels and tiller type (Fig. 3). Nevertheless, there were only significant N × T interactions in the area of SVB (Fig. 3D). Commonly, the number of LVB or SVB was the highest in the main stems, followed by early emerging tillers and late emerging tillers (Fig. 3A and B). For the same types of rice tillers, the number of LVB or SVB was significantly affected by N rates (*P *= 0.002); however, the differences caused by N treatment were lower than those caused by the tiller type (*P *< 0.001). Similar to the number of vascular bundles, the area of LVB or SVB was the highest in the main stem, followed by the early emerging tillers, with the lowest value for the late emerging tillers (Fig. 3C and D). The addition of N fertilizer significantly reduced the area of LVB. When the N rate was increased to N_247.5_, the area of LVB was decreased by 13.6 % in the main stem, 10.7 % in the early emerging tillers, and 25.1 % in the late emerging tillers, in comparison to N_0_. For the main and early tiller types, the application of N considerably increased the area of SVB in the main stem; however, its area in the late emerging tillers was significantly reduced.

**Figure 4:**
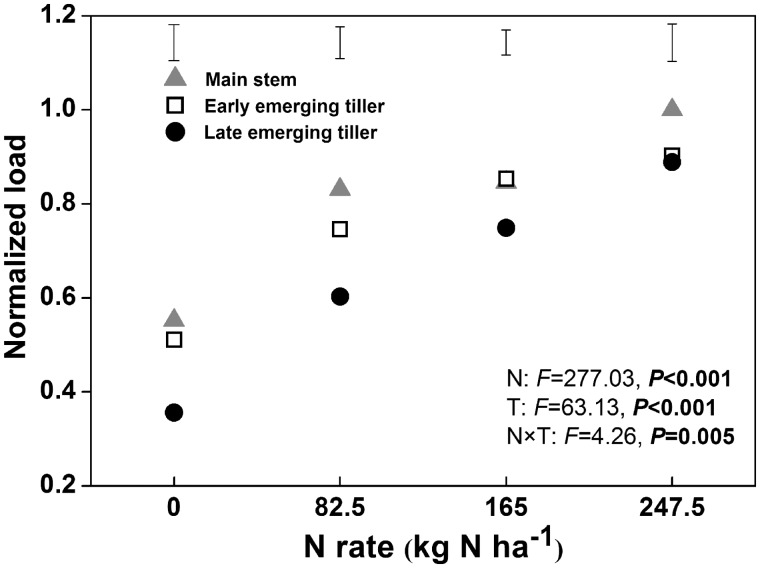
Normalization of transport load of the first internode of the stem for the different types of rice tillers grown under various N application rates in 2015. The df in Nitrogen (N), tiller type (T), N** **×** **T and error is 3, 2, 6 and 24, respectively. The vertical bars represent LSD at *P*** **=** **0.05.

To determine whether the flow between the source and sink was unrestricted and to compare N flow efficiencies between different rice tillers, the transport load was computed (Fig. 4). The application of N significantly increased the transport load, which indicated that the flow in no-N treatment was the most robust. The load in the late emerging tillers was less than in other types of tillers, but the difference was gradually reduced with higher N, and no obvious difference was observed under the N_247.5_ condition.

### Photosynthetic parameters and senescence process of the flag leaf

Nitrogen and tiller type significantly affected Φ_PSII_ and Φ_NPQ_; however, their interactive effect was not statistically significant for any of the variables measured (Table 6). The Φ_NO_ was significantly affected by tiller type in 2014; however, two-way ANOVA indicated that Φ_NO_ in 2015 was significantly affected by nitrogen rather than tiller type.

**Table 6.** Results of two-way analyses of variance (ANOVA) for analyses of differences in Φ_PSII_, Φ_NPQ_, Φ_NO_ and sucrose synthase activity to examine the effects of ‘nitrogen’ and ‘type’ for rice. SuSase, sucrose synthase. Values with P < 0.05 are in boldface.

Source	df	Φ_PSII_		Φ_NPQ_		Φ_NO_		SuSase activity	
		*F*	*P*	*F*	*P*	*F*	*P*	*F*	*P*
2014									
Nitrogen (N)	3	6.50	**0.002**	6.43	**0.002**	1.94	0.150	5.32	**0.006**
Type (T)	2	24.61	**<0.001**	7.56	**0.003**	4.95	**0.016**	35.58	**<0.001**
N×T	6	1.00	0.445	0.55	0.768	0.37	0.889	3.13	**0.021**
Error	24	–	–	–	–	–	–	–	–
2015									
N	3	7.826	**0.001**	11.21	**<0.001**	2.69	**0.069**	37.35	**<0.001**
T	2	10.44	**0.001**	9.04	**0.001**	2.30	0.122	62.01	**<0.001**
N×T	6	1.07	0.411	1.84	0.133	2.10	0.091	5.16	**0.002**
Error	24	–	–	–	–	–	–	–	–

**Figure 5:**
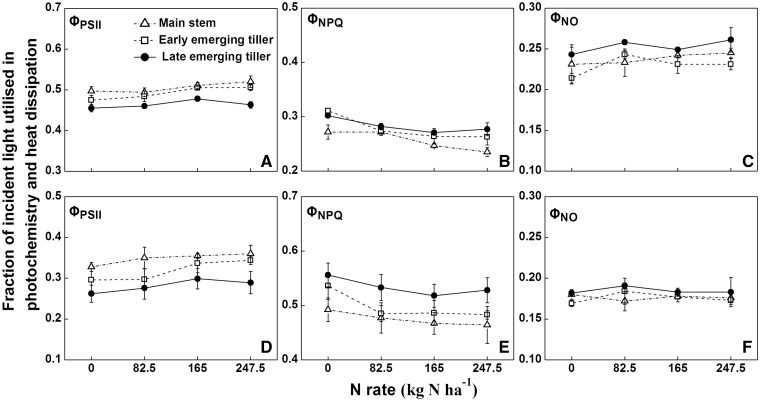
The quantum yields in the flag leaves of different types of rice tillers grown under different N applications in 2014 (A–C) and 2015 (D–F). Φ_PSII_, actual photochemistry efficiency; Φ_NPQ_, regulated thermal dissipation; Φ_NO_, constitutive non-photochemical energy dissipation. The error bars indicate the SD.

The fate of absorbed light energy in different types of tillers was evaluated in response to different N applications (Fig. 5). The Φ_PSII_ was the highest in the main stem, followed by early and late emerging tillers under all N levels in both years (Fig. 5A and D). The value of Φ_PSII_ in the same tiller type increased with increasing N levels. As the Φ_PSII_ in the late emerging tillers was always low, more absorbed light energy might be lost via thermal dissipation. The sequence of Φ_NPQ_ of different types of tillers was the reverse of that for Φ_PSII_ (Fig. 5B and E). A tendency for a decline in Φ_NPQ_ with increasing N applications was observed in all types of tiller. The proportion of non-photochemical energy dissipation (Φ_NO_) was lower than that of other light energy (Φ_PSII_ or Φ_NPQ_) (Fig. 5C and F). The results showed that the late emerging tillers possessed higher Φ_NO_ than the main stem or early emerging tillers, which might damage the photosynthetic system and accelerate the plant senescence process.

**Figure 6:**
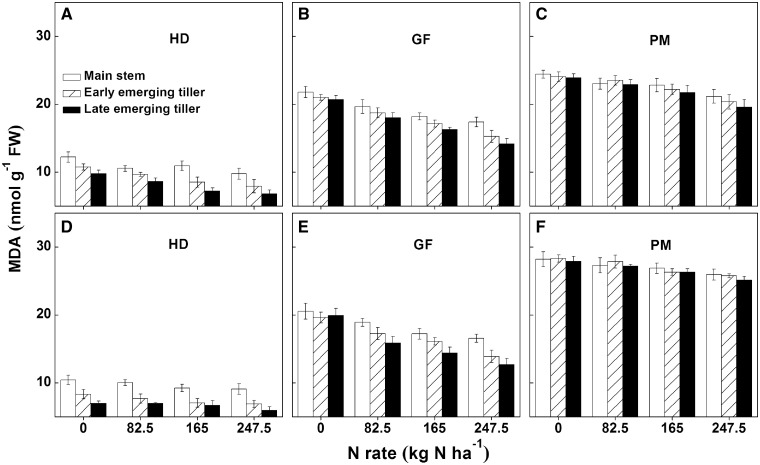
Lipid peroxidation rate (MDA content) in the flag leaves of different types of rice tillers at various stages grown under different N applications in 2014 (A–C) and 2015 (D–F). The error bars indicate the SD. HD, heading stage; GF, grain filling stage; PM, plant maturity stage.

Plant senescence, recognized as an increased concentration of MDA in the flag leaf, was low at the heading stage, and the highest at maturity in all types of tillers in both years (Fig. 6). The MDA concentration decreased with increasing N rates at similar stages of development. Under the same N levels, the MDA concentration was the highest in the main stems, followed by early and late emerging tillers, and the differences among tiller types were only significant at the heading stage in both years. However, such differences were gradually reduced with rice senescence; the MDA concentration was statistically similar in all types of tillers at maturity.

### Sucrose synthase activity in the grain

**Figure 7:**
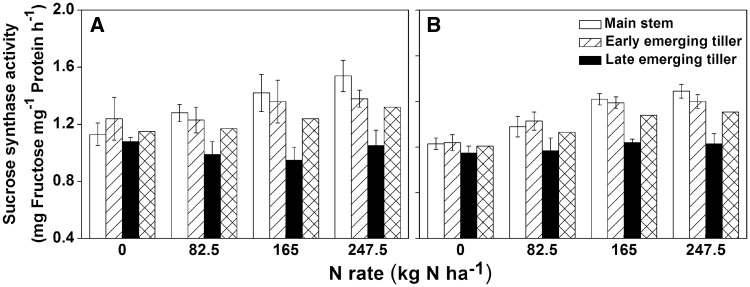
Sucrose synthase activity in the grains of different types of tillers at maturity grown under different N applications in 2014 (A) and 2015 (B). The error bars indicate the SD.

Sucrose synthase (in the cleavage direction; SuSase) in grains is the initial rate-limiting enzyme in the sucrose-to-starch pathway (Keeling *et al.* 1988). Two-way ANOVA revealed that the SuSase activity of grains was significantly affected by N application rate, tiller type and their interactive effect in 2014 and 2015 (Table 6). The results showed that SuSase activity in the late emerging tillers was significantly lower than that in the main stem or early emerging tillers under all N application treatments, except for no-N treatment (Fig. 7). However, there was no significant difference between the main stem and early emerging tillers in either year regarding the SuSase of grains. The SuSase activity increased with higher N rates within the same tiller type, except in the late emerging tillers.

## Discussion

### Effects of N rates on different types of tillers

The application of N fertilizer may increase the number of productive tillers (Budhar and Palaniappan 1996); however, not every tiller contributes similarly to high productivity (Sahu *et al.* 2004). In the present study, tillers were segregated as early and late emerging tillers, according to their time of emergence. Although the number of late emerging tillers was close to the number of early emerging tillers, the yield contribution of the late emerging tillers was significantly lower than that of the early emerging tillers (Table 5). That was because the yield of the late emerging tillers was typically lower than that of the early emerging tillers, with lower numbers of spikelets per panicle and grain filling percentage being the primary reasons for the low production of the late emerging tillers (Table 5). The yield contribution of early emerging tillers remained stable at ∼50 % under all N application rates, whereas the late emerging tillers could attain ∼30 % following application of sufficient N (Table 5). The yield contributions of the late emerging tillers became more important with higher N application rates; however, a significant yield gap existed between the early and late emerging tillers. Hence, it was necessary to study the limiting factors of late emerging tillers by comparing the physiological differences between these and the main stems and early emerging tillers.

Previous studies have reached different conclusions on yield-related parameters (number of spikelets per panicle, grain filling percentage, and grain weight), which either increased (Jian *et al.* 2014), were not affected (Mandana *et al.* 2014), or even decreased (Li *et al.* 2014), following the application of N fertilizer. These phenomena might be explained by our experimental results. Increasing N fertilizer not only improved the traits of main stems and early emerging tillers but also led to the production of a large number of low-yield late emerging tillers. Since yield components reflect the average of all tiller traits, inferior panicles might mask the effects of improved panicle characteristics of main stems and early emerging tillers.

### Effects of N rates on N flow efficiencies in different types of tillers

NHI is very useful for measuring N partitioning in rice plants, as it indicates the retranslocation efficiencies of absorbed N from straw to grain (Fageria 2014). Previous field studies have shown that the NHI in rice declined with increasing N rates, and that additional nutrients were intercepted by the straw (Chen *et al.* 2014; Jian *et al.* 2014); similar results were also observed in our experiments (Fig. 2). The differences in NHI between late emerging tillers and early emerging tillers gradually increased with increasing N rates (Fig. 2). This might be caused by the short growth duration of late emerging tillers, delayed emergence (the last productive tiller initiated ∼20 days later than the first productive tiller in the present study) and premature senescence (rapid malondialdehyde accumulation during filling stage; Fig. 6) which provided less time for N nutrients to be transported into grains. As the first internode at the top of the stem provided the only conduit for the transfer of nutrients from vegetative organs to grains, its development determined grain yield and N accumulation (Scofield *et al.* 2009). For the same types of rice tillers, the application of N fertilizer increased the amount and rate of N accumulation within the grain (Fig. 1), while it reduced the area of VB of the first internode (Fig. 3C and D). Unfortunately, neither N accumulation nor VB area could be employed to determine the level of transport efficiency; thus, a new ‘transport load’ (G/VB area) concept was utilized to represent the N flow efficiency response to N rate and tiller type. In the present study, the load was much higher under increased N treatments than under low N treatments (Fig. 4). The results indicated that the VB area was sufficient for N transport under low N conditions.

Furthermore, the difference in the transport load between the late emerging tillers and other types of tillers was reduced with increasing N rates (Fig. 4). The rapid increase in the load of late emerging tillers might be related to low irradiance, as shading promoted the transport of assimilation from the culm and sheath into the panicle (Yoshida 1972). In the rice field, as tiller development is asynchronous, early emerging tillers pre-empt the uppermost light source and shade late emerging tillers, and the high N application increased the shading effect on the late emerging tillers. It has been suggested that shading might be a unique factor (changes in N accumulation and VB area were the common factors for the three types of rice tillers) causing the late emerging tillers to increase the transport load. In addition, our results indicated that N flow was more efficient in the late emerging tillers than that of the main stem and early emerging tillers under all N treatments except for N_247.5_ (Fig. 4). However, the NHI in late emerging tillers was always less than that of the others under any N rate (Fig. 2). Although the emergence of the late emerging tillers obviously followed the early emerging tillers, their grain N accumulation was hindered at almost the same time (Fig. 1). Therefore, a brief filling period might be the primary factor that influences the NHI in late emerging tillers. 

### Analysis of delaying harvest time to enhance grain yield of late emerging tillers

In the field, the grains of the late emerging tillers were greener than early emerging tillers at harvest time, particularly under excess N conditions; thus it remains poorly understood whether it might be of value to wait for the ripening of these tillers (grains turn ‘yellow’). In the present study, the MDA concentration in different types of tillers gradually became similar toward maturity, and late emerging tillers showed a high senescence rate (Fig. 6). Kariali *et al.* (2012) also reported that rice tiller maturation was synchronous, meanwhile, the MDA content within different tillers was consistent from booting to maturity. Deficiency of endogenous cytokinin accelerates the production rate of intrinsic ethylene in the late emerging tillers and thus expedites their senescence (Kariali and Mohapatra 2007). In our experiments, more of the light energy absorbed by PSII was lost as expressed in the higher Φ_NO_ (constitutive non-photochemical energy dissipation) in late emerging tillers (Fig. 5C and F). A high Φ_NO_ indicates that excess absorbed light may not be consumed by photochemistry and regulated thermal dissipation, which may damage the photosynthetic apparatus (Kramer *et al.* 2004). Thus, heterogeneous energy distribution could be a further cause for the premature senescence of late emerging tillers. In addition, the grain activity of SuSase (in cleavage direction) in late emerging tillers was less than that of the main stem and early emerging tillers at the harvest stage, and high N applications increased the difference between them (Fig. 7). These results indicated that the degree of senescence of late emerging tillers was similar to that of the early emerging tillers at the harvest stage, therefore, delaying harvest time would not enhance grain yields in late emerging tillers, and could increase the shattering risk of mature grains in the main stems and early emerging tillers.

## Conclusions

The present study demonstrated that the number and contributions of late emerging tillers to rice grain yields were improved with increased N levels. However, the number of spikelets per panicle and grain filling of late emerging tillers were significantly lower than that of the main stem or early emerging tillers at all N levels. Cumulatively, the inappropriate light distribution in PSII and the premature aging of flag leaves led to insufficient resources for late emerging tillers. The low activity of SuSase and number of spikelets per panicle were the sources of inadequacies in grain sink activities and storage sites. In future, investigation of the physiological constraints associated with the low grain yield of late emerging rice tillers would likely be an effective approach to further enhance rice yields.

## Sources of Funding

This work was supported by the Special Fund for Agro-scientific Research in the Public Interest of China (grant no. 201503123), and the Fundamental Research Funds for the Central Universities (grant no. 2662015PY135).

## Contributions by Authors

Y.W., J.L. and X.L. conceived the idea, Y.W., C.G., S.W., S.H. and R.C. conducted the experiment, Y.W., J.L. and T.R. analysed the results, Y.W., X.L. and S.H. wrote the paper.

## Conflict of Interest Statement

None declared.
